# Indications for pancreaticoduodenectomy affected postoperative outcomes in octogenarians

**DOI:** 10.1002/ags3.12395

**Published:** 2020-08-31

**Authors:** Takehiro Okabayashi, Kenta Sui, Takahiro Murokawa, Jiro Kimura, Jun Iwata, Sojiro Morita, Tatsuo Iiyama, Yasuhiro Shimada

**Affiliations:** ^1^ Department of Gastroenterological Surgery Kochi Health Sciences Center Kochi Japan; ^2^ Department of Diagnostic Pathology Kochi Health Sciences Center Kochi Japan; ^3^ Department of Radiology Kochi Health Sciences Center Kochi Japan; ^4^ Department of Biostatistics National Center for Global Health and Medicine Shinjuku‐ku Japan; ^5^ Department of Clinical Oncology Kochi Health Sciences Center Kochi Japan

**Keywords:** elderly, octogenarian, outcome, pancreaticoduodenectomy, prognosis

## Abstract

**Aims:**

The safety and efficacy of pancreaticoduodenectomy (PD) in patients over the age of 80 years remain controversial. We aimed to examine post‐PD outcomes and to determine the age limit for PD.

**Methods:**

Patients were divided into two subgroups: the younger (<80 years) group and octogenarian (≥80 years) group. We retrospectively evaluated the clinical benefit of PD for periampullary diseases in the younger and octogenarian groups, focusing on short‐ and long‐term outcomes.

**Results:**

From March 2005 to December 2018, 586 consecutive surgically curable patients with diagnosed periampullary diseases were studied, among whom 122 (20.8%) were ≥80 years old. The general preoperative physical condition (G8 screening, instrumental activities of daily living, and Charlson comorbidity index) and nutritional status were significantly worse in the octogenarian group. However, there were no significant differences between the younger and octogenarian groups in postoperative severe complication rates (34% vs 36%) or perioperative mortality rates (1.5% vs 0.0%). We observed significantly poorer 3‐, 5‐, and 10‐year overall survivals in the octogenarian group than in the younger group (*P* = .007). In the younger group, the main cause of death (89.6%) was cancer recurrence. However, only 60% of patients in the octogenarian group developed and died from cancer recurrence. Increased neutrophilic/lymphocyte ratio and elevated Controlling Nutritional Status score were associated with worse outcomes.

**Conclusions:**

It is important to carefully determine the indication for PD in octogenarian patients with periampullary diseases, although patient age over 80 years should not be a contraindication for PD.

## Graphical abstract

The safety and efficacy of pancreaticoduodenectomy (PD) in patients over the age of 80 years remain controversial. Although our findings showed that the general preoperative condition and nutritional status were significantly worse in the octogenarian group, there were no significant differences between both groups regarding postoperative severe complication rates or perioperative mortality rates. The instrumental activities of daily living (IADL) scale in octogenarian patients might be preserved enough high functioning, although IADL scale in the octogenarian group was significantly lower than that in the younger group. The 3‐, 5‐, and 10‐year overall survival were significantly worse in the younger group than in the octogenarian group. In the younger group, the main cause of death (89.6%) was cancer recurrence. However, only 60% of patients in the octogenarian group developed and died from cancer recurrence.

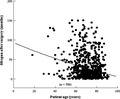

## 1 INTRODUCTION

Opportunities to perform highly invasive surgeries for the elderly and extend the average life span have increased worldwide by progress of safe operation management. The incidence of various cancers has increased and continues to increase as the mean population age rises in Western countries, as reported recently by the Japanese Ministry of Health, Labour, and Welfare.^1^, ^2^

Difficult operations are associated with a high rate of complications, but for pancreatoduodenectomy (PD), safety has greatly improved over the past 30 years for operations for pancreatic fistula, delayed gastric emptying, postoperative abdominal hemorrhage, intra‐abdominal abscess, and sepsis.^3^, ^4^ Because elderly patients are more likely to have comorbidities compared to young patients, effective PD strategies are essential. Furthermore, elderly people are more easily affected by perioperative adverse events.^5^ In recent years, improvements in operative and anesthetic techniques, regionalization to high‐volume centers, implementation of standardized recovery pathways, and better understanding and management of common complications have contributed to markedly improve short‐term outcomes after PD for the elderly.^6^, ^7^ With regard to the safety and effectiveness of complicated surgical resections in patients 80 years of age or older, only a few studies have reported the factors that are associated with poor disease prognoses and surgical outcomes. Furthermore, few studies have evaluated the relative advantages of surgical resection in patients 80 years of age or older with regards to operation outcomes.^8^, ^9^

Therefore, we retrospectively examined post‐PD outcomes in patients of various ages at our own institution. We also evaluated whether there was an age limit in performing PD.

## 2 METHODS

### 2.1 Patients

We retrospectively reviewed the surgical pathology database of the Kochi Health Science Center to identify patients who underwent pancreatectomies between April 2005 and December 2018. Patients who underwent PD during the study period were selected as the study cohort. The file system of the Medicare provider was used to distinguish the inpatient hospitalization requests of the Kochi Health Science Center. Statements that were either from the outpatient or submitted by a non‐facilities provider were identified using a standard outpatient file analysis system of the Kochi Health Science Center. Physical status and preoperative laboratory values were obtained within two weeks prior to the initiation of surgery. The American Society of Anesthesiologists (ASA) physical status classification system, G8 screening tool, instrumental activities of daily living (IADL), and Charlson comorbidity index (CCI) were available for all patients in our series.^10^, ^11^, ^12^, ^13^ The body mass index (BMI) was calculated by dividing the body weight in kilograms by the square of the height in meters. The prognostic nutritional index (PNI) was calculated based on the serum albumin and total lymphocyte count, using the following equation: PNI = 10 × serum albumin (g/dL) + 0.005 × total lymphocyte count (/mL).^14^ The Controlling Nutritional Status (CONUT) score is an index calculated from the following factors: serum albumin concentration, total peripheral lymphocyte count, and total cholesterol concentration.^15^ Our criteria, based on the proper selection of octogenarian patients with pancreatic cancer, were as follows: patients with (a) no severe comorbidities, (b) no cognitive impairment, (c) preserved preoperative functional status, (d) the expectation that the prognosis would be extended if surgical resection with intent‐to‐cure could be performed, compared to when surgical treatment was not performed, and (e) the desire for a cure for the pancreatic cancer.^16^ The institution's ERAS protocol for perioperative care of PD patients was developed based on guidelines by the ERAS society published in 2012.^17^ Our department followed the prognosis of each case and obtained accurate details of the outcomes. The study was approved by the ethics committee of Kochi Health Sciences Center. All patients provided written informed consent.

### 2.2 Assessment

After curative surgical resection, patients were divided into two groups: a younger group (<80 years of age) and an octogenarian group (80 years of age or older). The primary outcomes of this study were the rate of postoperative complication and overall survival in patients with PD, comparing the younger and octogenarian groups. Postoperative morbidities, including postoperative intra‐abdominal hemorrhage, intra‐abdominal abscess formation, postoperative ileus, pneumonia, delirium, pancreatic fistula, and delayed gastric emptying, were defined according to both the classification system of the International Study Group of Pancreatic Surgery (ISGPF) and the Clavien‐Dindo (C‐D) classification of surgical complications.^18^, ^19^ Overall survival was calculated from the date of surgery for periampullary diseases until the date of death in patients with or without recurrent disease. Postoperative complications and recurrence were examined by abdominal ultrasonography and/or computed tomography. Secondary outcomes included causes of early and late morbidity. We evaluated postoperative cholangitis and gastrointestinal bleeding. Cholangitis was defined according to the TG13 diagnostic guidelines and an acute cholangitis severity rating system after the operation.^20^ Known confounding variables included BMI, C‐reactive protein/albumin (CRP/Alb) ratio, platelet/lymphocyte ratio (PLR), and neutrophilic/lymphocyte ratio (NLR). The PNI variable represented the influence of the physio‐biological value before treatment, with respect to the continuous variable of OS. We used a Cox proportion hazard model, after regulating PLR and CONUT score. Patient‐specific mortality was categorized as early if it occurred between 0 and 90 days after PD, intermediate if it occurred from 91 to 365 days, and late if it occurred after 365 days. Causes of mortality were categorized as: (a) in‐hospital death due to PD complications; (b) cancer recurrence; (c) natural death without cancerous recurrence; (d) cancer death by newly developed malignancy; (e) multisystem organ failure (MSOF) secondary to aspiration, myocardial infarction, or renal dysfunction; and (f) suicide.

### 2.3 Statistical analysis

The data in this study were prospectively collected and was retrospectively analyzed by a biostatistician (TI). Survival curves were generated using the Kaplan‐Meier method and compared using the log‐rank test. Patients alive as of December 2018 were censored at the time of follow‐up. Qualitative variables were compared using the chi‐square test or Fisher's test, while quantitative variables were analyzed using Student's *t* test or a nonparametric test, and survival data were determined using a stratified log‐rank test. G8 screening tool, IADL score, and CCI were done using Mann‐Whitney *U* test. We made an addressee receiver operating characteristic curve, and the discrimination of the logistic model equation was decided by calculating a coincident indicator. A covariate‐adjusted restricted cubic spline regression analysis with three knots was performed to plot the survival after PD by age and to identify the age at which the survival after PD substantially decreased. This was done to determine the relationship between survival after PD and patient age. Differences in proportions were evaluated by Pearson's chi‐square test. All tests were two‐sided, with a *P*‐value of <.05 considered to indicate statistical significance. All analyses were performed using SPSS^®^ (SPSS).

## 3 RESULTS

### 3.1 Patients' demographics

From March 2005 to December 2018, 586 consecutive patients with periampullary disease who could be treated by intent‐to‐cure surgically were diagnosed by the Department of Gastroenterological Surgery at the Kochi Health Sciences Center. The preoperative patient characteristics are shown in detail in Table 1. Of these patients, 342 were men and 244 were women, ranging in age from 18 to 91 (median 71) years, with 122 patients (20.8%) aged 80 years or older. The rate of surgically treated female patients with PD in the octogenarian group (51.6%) was significantly greater than that in the younger group (39.0%) (*P* = .012). The BMI was significantly higher in the younger group than in the octogenarian group (median, 22.3 vs 21.4 kg/m^2^ *P* = .002) (Table 1). The prevalence of a previous medical history of concurrent diseases, such as ischemic heart disease, respiratory disease, cerebral infarction, chronic liver diseases, and/or diabetes mellitus, was similar between the groups. The octogenarian group showed a significantly worse general condition preoperatively than the younger group, according to the ASA physical status classification system (Table 1). Expectedly, the preoperative value of G8 screening tool in the octogenarian group was significantly reduced than that in the younger group (Table 1). And also, the baseline of IADL score and CCI in octogenarian group were significantly higher compared to those in younger group (Table 1). Preoperative serological C‐reactive protein/albumin ratio, and platelet/lymphocyte ratio did not differ between the groups, although the median hemoglobin count, PNI level, and CONUT score were significantly lower and the median NLR was significantly increased in the octogenarian group than in the younger group (Table 1).

**Table 1:** Patient demographics

Characteristics	Younger	Octogenarian	*P* value
	(n = 464)	(n = 122)	
Age (median, range)	68 (18‐79)	83 (80‐91)	<.001
Gender (male/female)	283/181	59/63	.012
Body mass index (median, range)	22.3 (14.4‐37.6)	21.4 (15.3‐34.2)	.002
Concurrent diseases (%)			
Ischemic heart disease	217 (46.8)	69 (56.6)	.054
Respiratory disease	53 (11.4)	19 (15.6)	.214
Cerebral infarction	50 (10.8)	18 (14.8)	.222
Chronic liver diseases	38 (8.2)	9 (7.4)	.915
Diabetes mellitus	140 (30.2)	31 (25.4)	.303
ASA‐PS (1/2/3)	56/326/82	0/80/42	.001
G8 screening tool (median, range)	11 (6‐16)	9 (5‐15)	<.001
IADL score (median, range)	8 (6‐8)	8 (6‐8)	<.001
CCI (median, range)	5 (0‐9)	7 (4‐10)	<.001
Preoperative nutritional status (median, range)			
Hemoglobin (g/dL)	13.0 (6.2‐16.9)	11.8 (7.3‐16.2)	.001
C‐reactive/albumin	0.03 (0.00‐5.60)	0.05 (0.00‐2.96)	.719
Platelet/lymphocyte ratio	14.8 (3.8‐175.5)	14.8 (3.5‐60.0)	.580
Neutrophil/lymphocyte ratio	2.3 (0.6‐19.5)	2.8 (0.5‐28.5)	.004
Prognostic nutritional index	47.9 (27.0‐65.1)	44.8 (32.0‐65.3)	.001
CONUT score	2 (0‐9)	3 (0‐9)	.001

Abbreviations: ASA‐PS, American Society of Anesthesiologists physical status; CCI, Charlson comorbidity index; CONUT, controlling nutritional status; IADL, instrumental activities of daily living by Lawton MP.

### 3.2 Surgery‐related characteristics

Surgery‐related demographics are represented in Table 2. Operation time was significantly shorter in the octogenarian group than in the younger group (median, 279 minutes in the younger group and 248 minutes in the octogenarian group, *P* = .001). Blood loss was not significantly different between the groups (median, 390 mL in the younger group and 320 mL in the octogenarian group; Table 2). The rate of porto‐mesenteric vein resection (PVR) was significantly different between the groups. In the younger group, 133 patients underwent (28.7%) PVR, while in the octogenarian group, only 14 patients (11.5%) underwent PVR (*P* = .001).

**Table 2:** Surgery‐related demographics

Characteristics	Younger	Octogenarian	Octogenarian		*P* value^a^	*P* value^b^
			Early period	Late period		
	(n = 464)	(n = 122)	(n = 45)	(n = 77)		
Operation time (min, median [range])	279 (153‐817)	248 (146‐822)	247 (146‐410)	248 (152‐822)	.001	.843
Blood loss volume (mL, median [range])	390 (20‐15 017)	320 (15‐15 330)	350 (20‐2990)	320 (15‐15 330)	.600	.687
Porto‐mesenteric vein resection (%)	133 (28.7)	14 (11.5)	6 (13.3)	8 (10.4)	.001	.843
Postoperative morbidities (%)						
Intra‐abdominal hemorrahge	15 (3.3)	2 (1.6)	1 (2.2)	1 (1.3)	.529	.725
Intra‐abdominal abscess formation	10 (2.2)	3 (2.5)	0 (0.0)	3 (3.9)	.887	.462
Postoperative ileus	1 (0.0)	1 (0.8)	0 (0.0)	1 (1.3)	.884	.784
Pneumonia	6 (1.3)	1 (0.8)	0 (0.0)	1 (1.3)	.968	.784
Delirium	15 (3.3)	29 (23.8)	12 (26.7)	17 (22.1)	.001	.566
Pancreatic fistula (A/B/C)	67/129/12	23/36/1	8/12/0	15/24/1	.584	.922
Delayed gastric emptying (grade B) (%)	28 (6.0)	4 (3.3)	1 (2.2)	3 (3.9)	.333	.979
C‐D classification (I/II/III/IV/V)	80/44/150/2/7	27/9/44/0/0	14/2/15/0/0	13/7/29/0/0	.535	.713
Hospital stay (days, median [range])	23 (8‐138)	22 (13‐152)	24 (14‐76)	20 (13‐152)	.732	.405
Pathologic demographics (%)						
Pancreatic neoplasms	294 (63.4)	73 (59.8)	20 (44.4)	53 (68.8)	.078	.046
Biliary neoplasms	78 (16.8)	33 (27.0)	20 (44.4)	13 (16.9)		
Duodenal neoplasms	68 (14.7)	13 (10.7)	4 (9.0)	9 (11.7)		
Chronic pancreatitis	23 (5.0)	3 (2.5)	1 (2.2)	2 (2.6)		
Duodenal perforation	1 (0.1)	0 (0.0)	0 (0.0)	0 (0.0)		

Notes

The “early period group” composed of patients who were treated between 2005 and 2012 and the “late period group” composed of patients treated between 2013 and 2018.

Abbreviation: C‐D classification; Clavien‐Dindo classification.

^a^ Statistical analysis between younger and octogenarian group.

^b^ Statistical analysis between early period and late period in octogenarian patients.

We evaluated surgery‐related characteristics in octogenarian patients who were divided into two groups: the “early period group” composed of patients who were treated between 2005 and 2012, and the “late period group” composed of patients treated between 2013 and 2018. PD for octogenarian patients was more frequently performed in the late period (Table 2). There was a significant difference between the two groups in octogenarian patients in terms of pathologic demographics. Interestingly, however, surgical duration (median operative time: 247 minutes in the early period group vs 248 minutes in the late period group) and blood loss volumes (median blood loss volume: 350 mL in the early period group vs 320 mL in the late period group) did not differ significantly between the two groups in octogenarian patients who underwent PD (Table 2).

### 3.3 Primary outcomes

#### 3.3.1 Short‐term outcomes

There were seven in‐hospital deaths (1.5%) among the younger group and no in‐hospital deaths (0.0%) among the octogenarian patients. All seven patients died during the primary hospital stay (total mortality, 1.2%). All seven deaths followed a surgical complication and were caused either by pancreatic fistula‐related bleeding (n = 4) or sepsis‐related multiple organ failure (n = 3). When patients were stratified according to younger or octogenarian groups, no significant difference was found with regard to postoperative complications, including intra‐abdominal hemorrhage, abscess formation, postoperative ileus, or pneumonia (Table 2). Notably, the incidence of complications, including pancreas fistula and delayed gastric emptying, did not significantly differ after the operation (for patients with grade B/C pancreas fistula by ISGPS classification, 30.4% of the younger group vs 30.3% of the octogenarian group; and with regards to the delayed gastric emptying of patients with grade B, by the ISGPS classification, 6.0% of the younger group vs 3.3% of the octogenarian group). Furthermore, grade III and V complications according to the C‐D classification also did not significantly differ between groups (34.3% of the younger group vs 36.1% of the octogenarian group), with regards to intra‐abdominal morbidities. However, a significantly higher incidence of postoperative delirium was observed in the octogenarian group than in the younger group (23.8% vs 3.3%; *P* = .001). There was no significant difference between the two groups regarding the duration of postoperative hospital stay, and there were no surgery‐related readmissions for any of the patients enrolled in the present study (median, 23 days in the younger group vs 22 days in the octogenarian group; Table 2). The final diagnoses of periampullary diseases in the younger group included pancreatic neoplasms in 294 patients, biliary neoplasms in 78, duodenal neoplasms in six, and chronic pancreatitis in 23. The diagnoses in the octogenarian group revealed 73 patients with pancreatic neoplasms, 33 with biliary neoplasms, 13 with duodenal neoplasms, and three with chronic pancreatitis (Table 2). Among the pancreatic cancer patients, 14 patients (19.2%) in the octogenarian group received adjuvant therapy and adjuvant chemotherapy was administered in 164 patients (55.8%) in the younger group (*P* < .01).

#### 3.3.2 Long‐term outcomes

**FIGURE 1:**
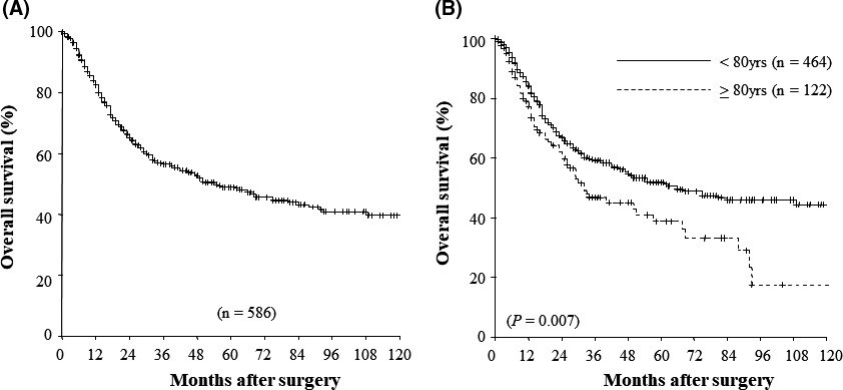
A, Overall 1‐, 3‐, 5‐, and 10‐y survival rates after surgery were 84.1%, 56.5%, 49.0%, and 39.6%, respectively. B, The overall survival curves of the two patient groups following whole pancreaticoduodenectomy at the time of data analysis. The 3‐, 5‐, and 10‐y survival rates for the younger vs octogenarian group were 59.5%, 51.7%, and 44.4%, respectively, vs 46.7%, 38.8%, and 17.5%, respectively (*P* = .007)

**FIGURE 2:**
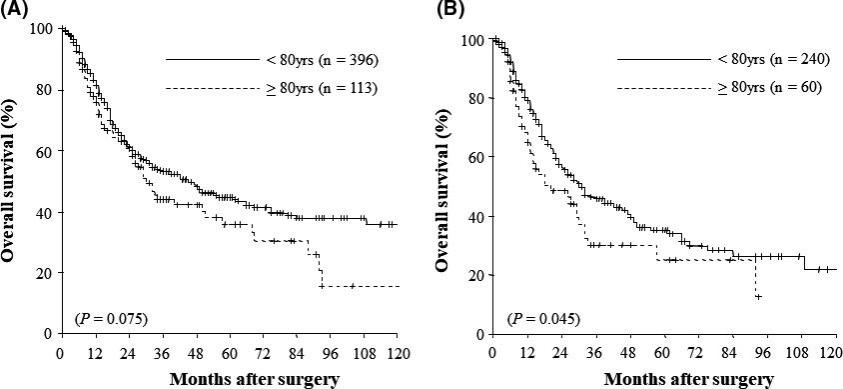
A, Overall, the 1‐, 3‐, and 5‐y survival rates in the younger patients with periampullary neoplasms after surgery were 83.1%, 53.6%, and 44.5%, respectively, while 1‐, 3‐, and 5‐y overall survival rates in octogenarian patients with periampullary neoplasms were 77.9%, 44.1%, and 35.9%, respectively. B, The overall survival in patients with pancreatic adenocarcinoma after curative resection with the intent to cure. The 3‐ and 5‐y survival rates for the younger vs octogenarian group were 46.5% and 35.1%, respectively, vs 30.3% and 25.2%, respectively (*P* = .045)

Patient follow‐up as of December 2018 ranged from 1 to 151 months with a median of 22.0 (mean 34.6) months. The analysis of overall survival was based on 245 deaths (41.8%) among the 586 patients, including 44 deaths in patients without recurrent disease (17 in the younger group and 27 in the octogenarian group) and seven cases of mortality induced by PD. Overall 1‐, 3‐, 5‐, and 10‐year survival rates after surgery were 84.1%, 56.5%, 49.0%, and 39.6%, respectively (Figure 1A). Figure 1B shows the overall survival curves of the two patient groups following whole PD at the time of data analysis. The 3‐, 5‐, and 10‐year survival rates for the younger vs octogenarian group were 59.5%, 51.7%, and 44.4%, respectively, vs 46.7%, 38.8%, and 17.5%, respectively. In the current study, we observed significantly poorer 3‐, 5‐, and 10‐year overall survival rates in the octogenarian group than in the younger group (Figure 1B) (*P* = .007). Overall, the 1‐, 3‐, and 5‐year survival rates in the younger patients with periampullary neoplasms after surgery were 83.1%, 53.6%, and 44.5%, respectively, while 1‐, 3‐, and 5‐year overall survival rates in octogenarian patients with periampullary neoplasms were 77.9%, 44.1%, and 35.9%, respectively (Figure 2A). There were no significant differences in overall survival rates between the groups (*P* = .075). Figure 2B represents the overall survival in patients with pancreatic adenocarcinoma after curative resection with the intent to cure. The 3‐ and 5‐year survival rates for the younger vs octogenarian group were 46.5% and 35.1%, respectively, vs 30.3% and 25.2%, respectively. In the current study, we observed significantly poorer 3‐ and 5‐year overall survival rates in octogenarian vs younger patients with pancreatic adenocarcinoma after PD (*P* = .045).

### 3.4 Secondary outcomes

**FIGURE 3:**
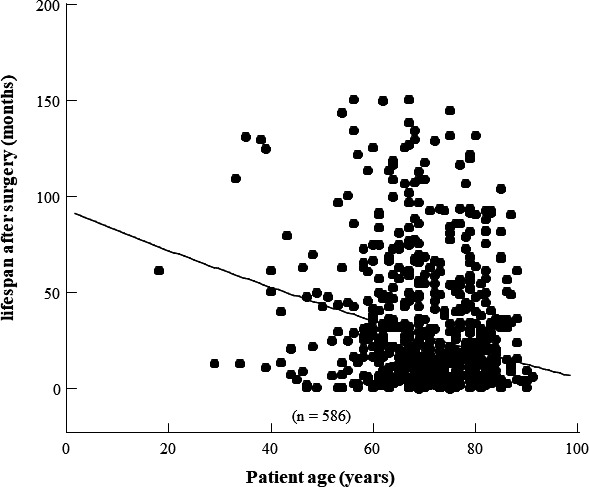
The lifespan volumetric trajectories after pancreaticoduodenectomy using covariate‐adjusted restricted cubic spline regression as well as the age at the time of surgery

All the mortalities after PD are shown in Table 3. Of 245 patients, 91 (37.1%) died within 1 year after PD. Thirteen patients (5.3%) experienced early mortality (0‐90 days postoperatively), 78 (31.8%) experienced intermediate mortality (91‐365 days postoperatively), and 154 (62.9%) experienced late mortality (over 366 days postoperatively). One‐year mortality was lower in patients with benign or premalignant diagnosis compared to patients with pancreatic adenocarcinoma or other malignancies (0.0% vs 27.3%, *P* < .001). In our series, 1‐year mortality was not significantly higher in the octogenarian group than in the younger group (41.3% vs 35.7%). Throughout the early, intermediate, and late postoperative periods, cancer recurrence in 163 patients (89.6%) was the most common cause of mortality in the younger group. Other causes of mortality after PD in the younger group were natural death in nine patients, newly developed cancer in four patients (one gastric cancer, one intrahepatic cholangiocarcinoma, one lung cancer, and one leukemia), and MSOF in six patients (three pneumonia, one myocardial infarction, one renal dysfunction, and one peritonitis due to colon perforation). In contrast, only 37 patients (58.7%) died of cancer recurrence after PD in the octogenarian group. The incidence of mortality due to cancer recurrence was significantly lower in the octogenarian group than in the younger group (*P* = .001). Other causes of death in the octogenarian group were natural death (n = 19), MSOF (n = 6; four pneumonia, two myocardial infarction), and suicide (n = 1). Newly developed cancer did not occur after PD in the octogenarian patients. Refractory cholangitis was observed in 35 patients (7.0%) between the intermediate and late period in this study. The incidence of long‐term cholangitis after PD was 4.0% (n = 5) among the octogenarian group and 6.5% (n = 30) among the younger group with no significant difference. No gastrointestinal hemorrhage occurred in the late period in the current series. The associations of physio‐biological characteristics with OS are detailed in Table 4. On multivariate analysis, increased NLR (hazard ratio [HR] = 1.48; 95% CI: 1.04‐2.03, *P* = .04), and elevated CONUT score (HR = 1.65; 95% CI: 1.12‐2.43; *P* = .01) were associated with worse outcomes (Table 4). Figure 3 shows the lifespan of volumetric trajectories after PD using covariate‐adjusted restricted cubic spline regression as well as the age at the time of surgery. When the age cutoff is 80 years at the time of surgery, the proposed life expectancy would be 24 months. However, if the age cutoff is increased to 85 years, with 27 patients older than 85 years, the proposed life expectancy would be only 18 months.

**Table 3:** Mortality after pancreaticoduodenectomy

Characteristics	Early (0‐90 d)	Intermediate (91‐365 d)	Late (over 366 d)
Younger	9	56	117
In‐hospital death	7	—	—
Gender			
Male/female	3/6	28/28	66/51
Diagnosis			
PDAC (without rec)	7 (3)	38 (3)	82 (5)
Other malignancy (without rec)	2 (1)	18 (2)	33 (3)
Premalignant	—	—	2
Benign	—	—	—
Octogenarian	4	22	37
In—hospital death	0	—	—
Gender			
Male/female	3/1	10/12	20/17
Diagnosis			
PDAC (without rec)	2 (1)	20 (5)	19 (7)
Other malignancy (without rec)	2 (0)	2 (1)	18 (12)
Premalignant	—	—	—
Benign	—	—	—

Abbreviation: PDAC, pancreatic adenocarcinoma.

**Table 4:** Association of overall survival with physio‐biologic characteristics in patients who underwent pancreaticoduodenectomy

Demographics		n	Univariate			Multivariate		
			MST	5 y, 10 y OS	*P*	HR	95% CI	*P*
BMI	>22	296	75	53.7%, 46.5%				
	<22	290	47	44.7%, 29.4%	.03	0.817	0.582‐1.146	.24
Hb (g/dL)	>11	199	66	51.4%, 40.7%				
	<11	387	45	44.1%, 31.2%	.18			
CRP/alb	<0.05	327	63	51.3%, 38.1%				
	>0.05	259	48	46.6%, 38.1%	.20			
PLR	<14.8	293	66	52.0%, 34.6%				
	>14.8	293	49	46.0%, 41.5%	.63			
NLR	<2.5	337	75	54.9%, 42.9%				
	>2.5	249	36	40.8%, 34.6%	.01	1.483	1.043‐2.028	.04
PNI	<40	91	42	47.8%, 34.9%				
	>40	495	56	49.3%, 38.0%	.63			
CONUT	<3	191	109	57.3%, 49.0%				
	>3	395	45	45.2%, 32.6%	.01	1.648	1.119‐2.426	.01

Abbreviations: CI, confidence interval; CONUT, controlling nutritional status; CRP/Alb, C‐reactive protein/albumin ratio; Hg, hemoglobin; HR, hazard ratio; MST, Median survival time (months after surgery); NLR, neutrophil/lymphocyte ratio; OS, overall survival; PLR, platelet/lymphocyte ratio; PNI, prognostic nutritional index.

## 4 DISCUSSION

This is the first report addressing how old patients with periampullary diseases may benefit from PD and how to assess the indication and age limitations of patients undergoing PD. In the current study, the remedial treatment achieved superior PD for patients with periampullary disease who were 80 years of age or older. Furthermore, a meaningful increase in postoperative complications was not found for patients aged 80 years old or over compared with that for those younger than 80 years. However, significantly more patients in the octogenarian group experienced postoperative delirium compared to the younger group, although this complication was not considered to be serious. Several publications over the past decade have reported on the outcomes of PD in patients aged 80 years and older compared to younger patients.^7^, ^9^, ^16^ Undoubtedly, the current study is a very pertinent topic considering our aging population and the current major question was which patients should be choosen as proper octogenarians for PD for periampullary diseases and how to do it. Obviously, because of improvements in preoperative diagnostic technology, surgical techniques, and perioperative care, patient treatment has dramatically ameliorated in recent years, thereby reducing the mortality rate and improving postoperative outcomes. Thus, the incidence of periampullary diseases to offer PD is increasing and will continue to increase as the average population age rises.

In general, age is not the primary consideration for surgical risk; performance status and comorbidities of the patient are more important factors than a patient's age. Geriatric assessment for elderly patients is essential to perform such a surgery with many complications. In our series, the preoperative physical status and concurrent comorbidities in octogenarian patients according to the G8 screening tool and CCI were significantly reduced compared to younger patients. However, from the viewpoint of functioning in the community setting, IADL scale in octogenarian patients might be preserved enough high functioning, although IADL scale score in the octogenarian group was significantly lower than that in the younger group. Overall, our results suggest that age is not a contraindication for PD and that surgical treatment for octogenarians with periampullary diseases according to our subjective criteria in addition to objective evaluation like G8 screening, IADL, and CCI could be of clinical benefit with acceptable long‐term survival results.^11^, ^12^, ^13^, ^21^, ^22^ The fact that the ranges of G8 and CCI did not differ greatly between the two groups is considered to be evidence that appropriate patient selection has been performed. This study could also contribute to better‐informed decision‐making for octogenarian patients and their families.

The immunonutritional and physical status is an important prognostic factor for perioperative outcomes.^23^ In our subgroup analysis, elevated CONUT score and increased NLR were extracted as independent risk factors for long‐term worse outcome after PD. Firstly, for a long time, serum albumin has been used as a substitute index for patient nourishment status. Low serum albumin levels are related to increased perioperative morbidity and mortality. However, there is a limitation to using serum albumin as the only nourishment state determinant, since albumin is a negative immediate that may become abnormal in states other than malnutrition, such as in chronic diseases.^24^ Therefore, preoperative nutritional status was evaluated using CONUT score, and the preoperative nutritional status of octogenarian patients with PD was significantly lower compared to that of the younger patients. Secondly, the NLR has emerged as an attractive proxy to characterize systemic immune‐inflammatory status. A recent study showed that NLR is a strong prognostic indicator for patients suffering from various diseases. Further, NLR has also been associated with poor clinical outcomes in a variety of diseases including myocardial infarction, coronary artery disease, atherosclerosis, and chronic obstructive pulmonary disease.^25^ In our series, the NLR was significantly increased in the octogenarian group than in the younger group. Our results might suggest that the preoperative immunonutritional status of octogenarian patients predicts long‐term survival following PD for periampullary diseases.^18^, ^26^ This assessment is especially important in order to have an accurate discussion regarding the risks and benefits of PD with patients who have periampullary diseases prior to the decision to operate.

The understanding of the relationship between age and complications may be insufficient to counsel patients and it has remained unclear whether an age cutoff exists where the risk of both early and late complications, including mortality, significantly increases. For most patients (89.6%) in the younger group, the main cause of death was cancer recurrence or metastasis after PD for pancreatic adenocarcinoma. However, up to 60% of patients in the octogenarian group who underwent complete curative resection for periampullary malignant neoplasms developed and died from distant metastases. Patient selection as it relates to preoperative functional performance is important.^27^, ^28^ Natural death or MSOF was the second most common cause of octogenarian patient mortality in the postoperative period after PD. Whereas our series used the cutoff of 80 years as a “symbolic” age value relating to “old age” without a validation process, the proposed life expectancy in the octogenarian group would be 24 months after PD, when the age cutoff is 80 years at the time of surgery. If patients over 85 years old receive PD, the proposed life expectancy is <2 years. Therefore, it is important to carefully determine the indication for PD in octogenarian patients with periampullary diseases.

This study has several potential limitations. First, we acknowledge the presence of a selection bias, since patients included in our study were admitted for surgery at a single institute. Moreover, this study was also a retrospective cohort review of patients undergoing PD that included only patients who underwent a resection. Second, our study focused on the short‐ and long‐term outcomes in patients with periampullary diseases after PD; hence, it is important to consider the postoperative quality of life as a treatment outcome in addition to operative mortality and long‐term survival rates. This design inherently represents some selection bias, based on the selection of patients chosen to undergo pancreatectomy, particularly for the older than 80 years group. The current study seems to address a very pertinent topic in our aging population and the current major question aims to determine which octogenarians should benefit from PD for periampullary diseases and how to perform PD for such patients. Therefore, it is necessary to evaluate patient comorbidities, cognitive status, preoperative functional state, and frailty. Furthermore, patients who will benefit from surgical resection should also be chosen according to octogenarian periampullary disease. However, despite these limits, we focus on the importance of PD management in the current study of octogenarians, where treatment regimens were chosen based on patient characteristics. The final decision to perform an operation may depend on the patient’s preference, and our results may be presented to patients during informed consent.

In conclusion, our results suggest that age over 80 years should not be a contraindication for PD. Therefore, we are careful not to exaggerate our findings, and octogenarian patients should not be denied surgical opportunities when it is thought that the patient is an ideal candidate for the treatment and the agreement of the family is provided.

## DISCLOSURE

Funding: This work was supported by grants from the Kochi Organization for Medical Reformation and Renewal.

Conflicts of Interest: None declared.
